# Erector Spinae Block in Bariatric Surgery: a Systematic Review and Meta-Analysis of Randomized Controlled Trials

**DOI:** 10.1007/s11695-026-08935-4

**Published:** 2026-09-22

**Authors:** Giovanna Macanhã Scremin, Pedro Bicudo Bregion, Victor Kenzo Ivano, Gabriel José Souto Maior de França, Julia Bueno de Oliveira Alves, Luísa Zanelatto de Araujo, Pandora Eloa Oliveira Fonseca, Leonardo Halamy Pereira, João Gabriel Romero Braga, Everton Cazzo

**Affiliations:** 1 Pontifícia Universidade Católica do Paraná, Curitiba, Brazil; 2 State University of Campinas, Campinas, Brazil; 3 Federal University of Pernambuco, Recife, Brazil; 4 Federal University of Rio Grande do Sul, Porto Alegre, Brazil; 5 Federal University of Campina Grande, Campina Grande, Brazil; 6 Fluminense Federal University, Niterói, Brazil; 7 Fundação Padre Albino, Catanduva, Brazil

**Keywords:** Bariatric surgery, Obesity, Anesthesia and analgesia, Anesthesia recovery period, Infiltration anesthesia, Opioids, Postoperative pain

## Abstract

**Introduction:**

Obesity is a growing global health issue, and bariatric surgery offers effective weight loss outcomes. Postoperative pain management remains a challenge, with multimodal analgesia and regional blocks like the erector spinae plane (ESPB) showing promise. This review aims to evaluate the efficacy of the Erector Spinae Plane Block (ESPB) on key postoperative outcomes in bariatric surgery, specifically its impact on opioid consumption, pain management, and complication rates.

**Methods:**

We conducted a systematic review and meta-analysis following PRISMA guidelines. In January 2025, we conducted a comprehensive search across PubMed, Embase, and Cochrane databases for studies comparing erector spinae block in bariatric surgery. Data extraction was standardized, focusing on postoperative analgesic consumption, pain control, intraoperative opioid consumption, respiratory complications and PONV. Statistical analysis was performed using RevMan 9.0.0, with random-effects models. Heterogeneity was assessed using Cochran’s Q test and I² statistic.

**Results:**

The analysis included 9 randomized controlled trials (RCTs) with a total of 553 patients. ESP block significantly reduced total opioid consumption (MD -9.53, 95% CI -15.10 to -3.96, *p* = 0.0008), pain scores like NRS (MD -1.15, 95% CI -1.76 to -0.55, *p* = 0.0002). Despite heterogeneity across studies, ESPB was associated with reduced opioid use and better pain control. ESP block was associated with a significant reduction in VAS pain scores during the first 12 h after surgery, although substantial heterogeneity was observed across studies. Using the ESP block significantly reduced the incidence of postoperative nausea and vomiting, with consistent results across studies (I² = 0%). There was no significant difference in the incidence of respiratory complications between the study groups.

**Conclusion:**

ESPB is associated with significant reductions in postoperative pain scores and total opioid consumption, while no significant difference in respiratory complications was observed between the groups.

**Supplementary Information:**

The online version contains supplementary material available at https://doi.org/10.1007/s11695-026-08935-4.

## Introduction

 Obesity is a growing global public health issue. It is projected that the number of adults living with obesity will increase from 0.81 billion in 2020 to 1.53 billion by 2035 [1]. Among the most effective treatment strategies is bariatric surgery, which provides sustained weight loss when combined with lifestyle modifications [2].

Seeking the optimal surgical technique, minimizing invasiveness is also a key goal of modern bariatric care. Despite advances, postoperative pain control and opioid reduction still need improvement to avoid adverse effects such as nausea and vomiting, which can delay hospital discharge. Previous studies have explored the role of multimodal analgesia, especially anesthetic blocks like the transversus abdominis plane (TAP) block [3–5]. A recent systematic review about TAP block, including 11 randomized controlled trials (RCTs), showed a significant reduction in opioid consumption within the first 24 h after surgery, fewer patients requiring rescue analgesia, and significantly improved pain scores [3].

Other regional blocks have shown promising results. A recent RCT comparing the erector spinae plane block (ESPB) to the quadratus lumborum block (QLB) in laparoscopic colorectal surgery found that ESPB was associated with delayed need for rescue analgesia (280 ± 15.5 min vs. 260 ± 13.8 min) and reduced 24-hour nalbuphine consumption (24 ± 2.5 mg vs. 30.5 ± 1.55 mg) [6]. Further investigation into anesthetic techniques such as ESPB is necessary. Their integration into multimodal analgesia protocols holds potential for improving postoperative outcomes and reducing opioid consumption in bariatric surgery. This study aims to assess the effectiveness of ESPB in bariatric surgery for improving postoperative outcomes and reducing opioid consumption.

## Methods

We conducted a systematic review and meta-analysis following the PRISMA guidelines. In January 2025, a comprehensive search was performed in PubMed, Embase, and Cochrane databases. The review was registered in PROSPERO under the number [CRD420251026072].

### Search Strategy

The search strategy combined indexed terms (MeSH, Emtree) and keywords related to bariatric surgery and erector spinae plane (ESP) block. No language restrictions were applied. We used the following search strategy in the databases: (“bariatric surgery” OR “sleeve” OR “gastrectomy” OR “gastric bypass”) AND (“ultrasound-guided” OR “erector spinae plane block” OR “ESP block”) AND (randomized controlled trial). The complete search strategy for each database is provided in the Supplementary material.

### Eligibility Criteria

Studies were included if they met the following criteria: (1) patients undergoing bariatric surgery; (2) ultrasound-guided bilateral erector spinae plane (ESP) block as the intervention; (3) placebo (saline) or no intervention in the control group. If the study had three or more arms, such as control, ESBP and other ultrasound blocks, it was included. Conversely, studies without control groups, or those that do not describe ESBP were excluded.

**Fig. 1:**
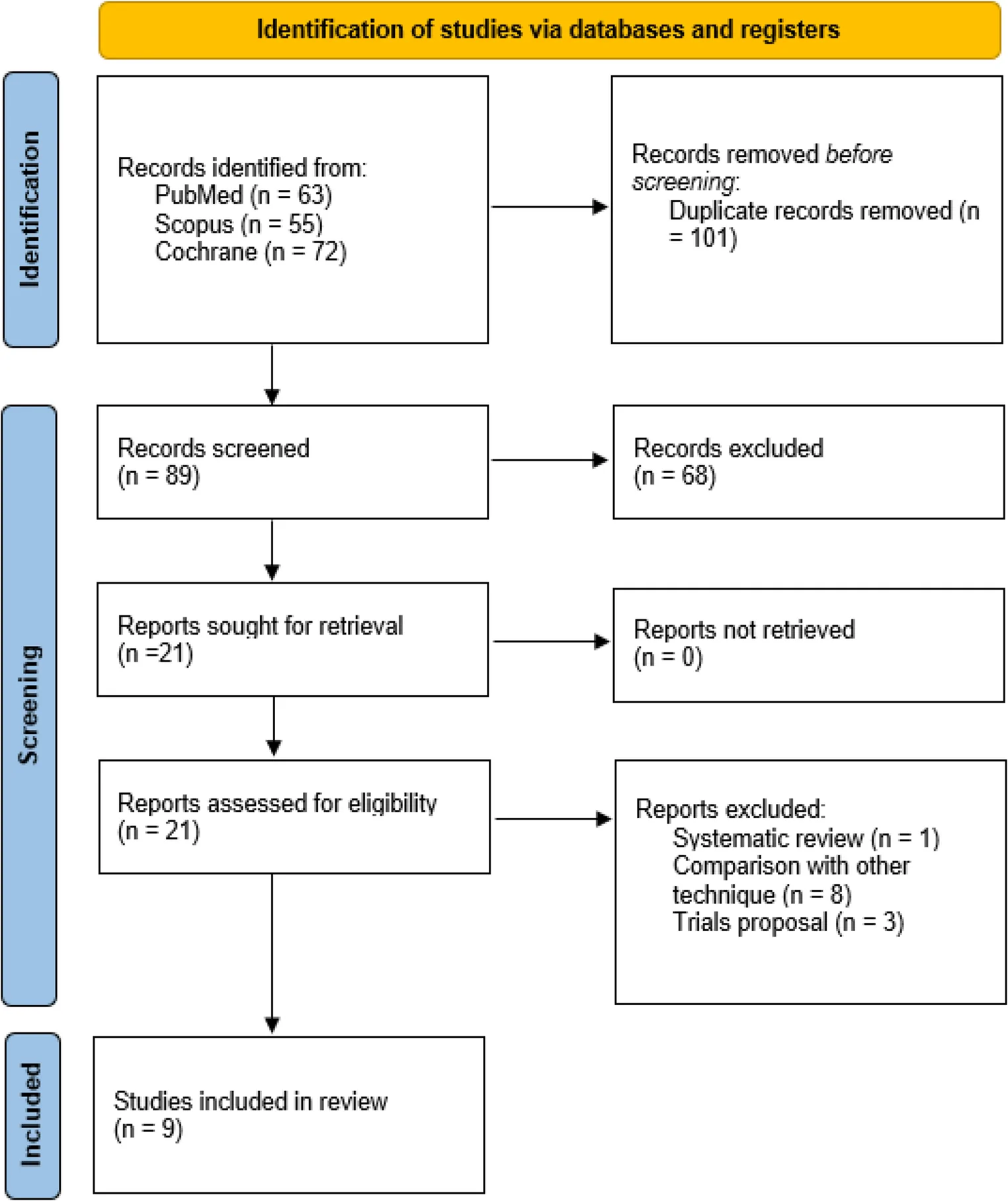
PRISMA flowchart

The study selection process was carried out independently by two reviewers (G.M.S and V.K) in two stages: first, by search strategy and study designs. The identified studies were imported into Rayyan software, and duplicates were removed. Two independent reviewers (G.M.S and L.Z.A) screened the titles and abstracts. Potentially eligible studies were assessed in full text, and disagreements were resolved by a third reviewer (P.E.O.F). The selection process is detailed in the PRISMA flowchart (Fig. 1). Additionally, we manually examined the reference lists of full-text review articles to identify other eligible studies.

The studies by Ashoor et al. [7] and Abdelhamid et al. [8] were the only two studies to compare two anesthetic techniques with a control group.Ashoor randomly assigned patients to 3 groups ESPB group, quadratus lumborum block (QLB) group, or the control group. Abdelhamid, randomly assigned patients to either one of three study groups on the day of surgery, ESPB, transversus abdominis plane (TAP) block or control group. For this meta-analysis, only the comparisons between ESPB and the control group were used.

### Data Extraction and Outcomes

We assessed postoperative analgesic consumption and effective postoperative pain control as primary outcomes. As secondary outcomes, we assessed intraoperative opioid consumption and respiratory complications, as well as postoperative nausea and vomiting (PONV) symptoms. Studies were excluded if they had an inadequate population, compared ESP block with another blocking technique, or involved open surgical procedures.

We extracted the data using a standardized form, including general information such as author, year, and population characteristics (age, sex, body mass index, and ASA score, type of surgery). We also extracted data on the evaluated outcomes, including pain assessment scores (visual analog scale - VAS score and numeric pain rating scale - NRS score), total opioid consumption, intraoperative opioid consumption, respiratory complications, and postoperative nausea and vomiting (PONV) symptoms.

For studies included in the review that did not report complete numerical data, but presented only graphical representations, data extraction was performed using the WebPlotDigitizer software (version 5.2, Ankit Rohatgi). Means and confidence intervals (or standard deviations, when available) were extracted directly from bar charts or line graphs provided in the original articles. The extraction process involved careful calibration of the graph axes to ensure the highest possible accuracy of the retrieved values. When necessary, the extracted data were converted into means and standard deviations according to the recommendations of the Cochrane Handbook for Systematic Reviews of Interventions.

### Statistical Analysis

The meta-analysis was conducted using RevMan 9.0.0 software. Effect measures were calculated using relative risk (RR) and mean difference (MD), with a 95% confidence interval (CI). A random-effects model was applied to account for the heterogeneity among the studies. Heterogeneity was assessed using Cochran’s Q test and the I² statistic, classified as low (< 25%), moderate (25–50%), or high (> 50%). Two authors used RoB 2: A revised Cochrane risk-of-bias tool for randomized trials. Publication bias was assessed through visual inspection of the funnel plot and Egger/Begg’s test, when appropriate. Sensitivity analysis was performed as subgroup analysis in cases of high heterogeneity.

## Results

### Study Characteristics

From the systematic search, we retrieved nine studies that all met the inclusion criteria for the final analysis. All the studies were randomized controlled trials, comprising a total of 553 patients. Figure 1 presents the PRISMA flowchart detailing the study selection process. Table 1 shows the details of the included studies published between 2020 and 2025.

**Table 1:** Characteristics of the studies included in the meta-analysis, including authors, year of publication, number of patients, body mass index (BMI), type of surgery and follow-up time

Study	Design	Number of patients		Female sex		Age years (mean ± standard deviation)		BMI kg/m² (mean ± standard deviation)		Type of surgery		Follow-up time
		ESPB	Control	ESPB	Control	ESPB	Control	ESPB	Control	Sleeve	Bypass	
Zengin et al., [9]	RCT	30	30	------	------	40.2 ± 12.2	39.4 ± 10.5	-----	------	------	------	24 h
Karaveli et al., [10]	RCT	20	20	19	17	40.05 ± 13.63	41.45 ± 12.41	43.25 ± 4.38	43.12 ± 3.39	40	0	24 h
Jinaworn et al., [11]	RCT	31	30	17	16	36.06 ± 9.41	35.80 ± 8.53	44.37 ± 8.51	44.32 ± 7.34	39	22	24 h
Toprak et al., [12]	RCT	40	40	32	32	37.60 ± 9.87	37.50 ± 10.15	46.25 ± 8.31	47.90 ± 15.44	27	53	48 h
Huda et al., [13]	RCT	25	25	6	16	-------------	--------------	--------	-------	50	0	24 h
Wang et al., [14]	RCT	76	75	66	67	32.8 ± 6.5	33.0 ± 6.4	37.6 ± 3.9	38.4 ± 5.9	151	0	48 h
Mostafa et al., [15]	RCT	30	30	26	25	38.80 ± 6.65	40.30 ± 7.86	50.37 ± 5.02	48.20 ± 4.33	41	19	24 h

*BMI* body mass index, *ESPB* erector spinae plane block

### Patient Characteristics

Table 1 provides a summary of the demographic characteristics of the patient populations across the studies. The age ranged from 32.8 to 41.45 years. The mean body mass index (BMI) ranged from 37.9 to 50.37. Regarding the type of surgery, 459 patients were submitted to sleeve gastrectomy, and 94 were submitted to bypass surgery. The follow-up time was from 24 to 48 h.

### Intraoperative Opioid Consumption

**Fig. 2:**
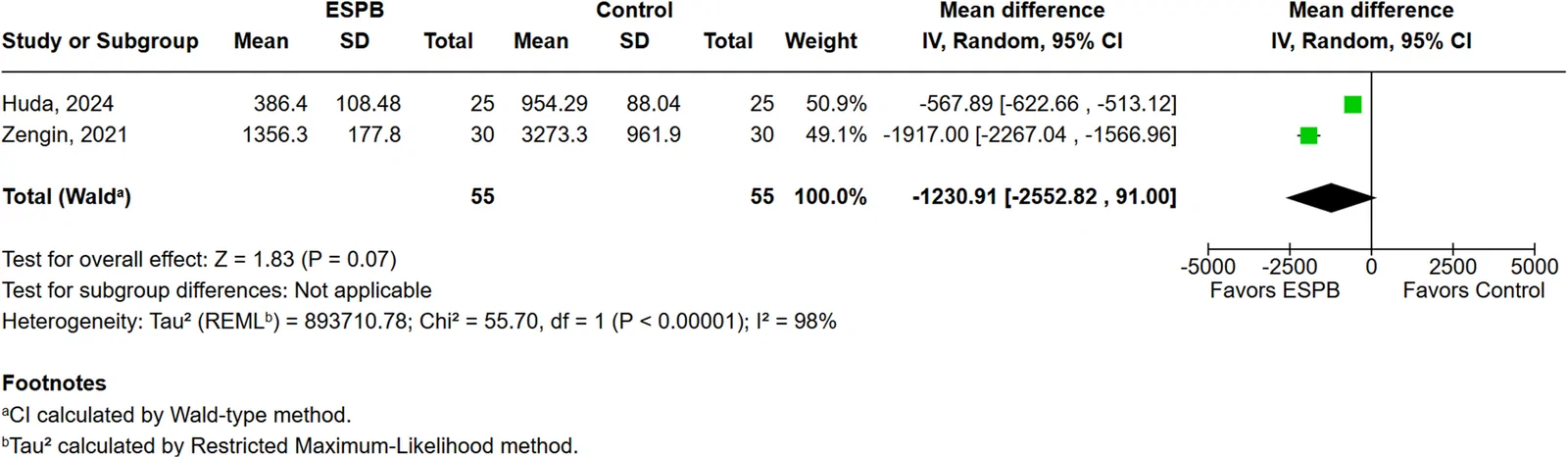
Intraoperative remifentanil consumption

In the analysis of intraoperative opioid use, we evaluated two distinct subgroups: remifentanil and fentanyl. There was a strong trend toward reduced remifentanil consumption in patients who received ESPB compared to controls (MD − 1230.91; 95% CI − 2552.82 to 91.00; *p* = 0.07) (Fig. 2). However, this outcome was derived from only two studies and should therefore be interpreted with caution, as the analysis is underpowered and demonstrated substantial heterogeneity (I² = 98%).

**Fig. 3:**
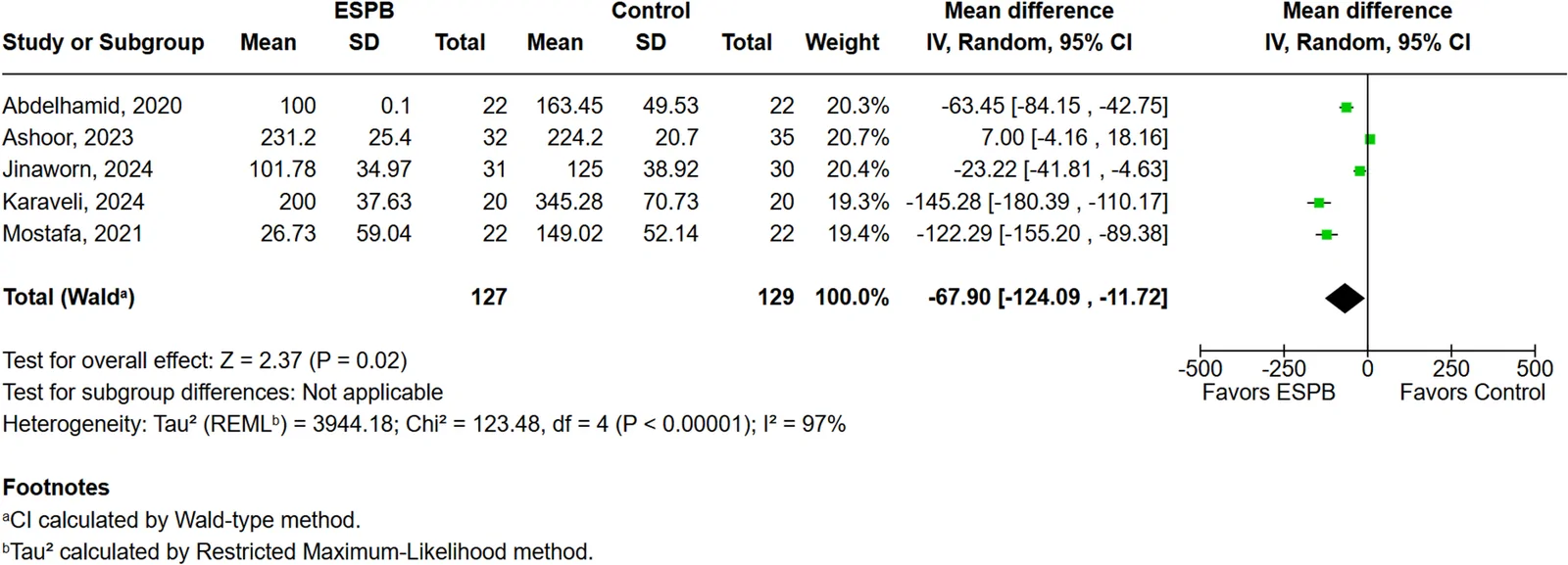
Intraoperative fentanyl consumption

Regarding intraoperative fentanyl consumption, we observed a significant reduction in ESPB group (MD − 67.90; 95% CI − 124.09 to −11.72; *p* = 0.02) (Fig. 3). Notably, sensitivity analysis did not reveal a single study as an outlier.

Abdelhamid et al. [8] reported intraoperative fentanyl consumption using median and interquartile range. In the ESPB group, all patients received the same standardized induction dose of 100 mcg, resulting in a median and interquartile range of 100–100 mcg. Only one patient required an additional dose during surgery, which did not alter the IQR due to rounding and sample size. Therefore, to enable inclusion of this study in the meta-analysis model, since a standard deviation (SD) of 0 would otherwise prevent appropriate weighting, a minimal SD value of 0.1 was imputed. The authors demonstrated that both TAP and ESPB groups were significantly superior to the control group in reducing intraoperative opioid requirements.

Intraoperative opioid use was generally guided by hemodynamic responses, with most studies administering additional doses in response to elevations in heart rate (HR) or mean arterial pressure (MAP) exceeding 20% from baseline. Studies utilizing fentanyl [7, 8, 10, 15] adopted similar protocols, delivering bolus doses ranging from 0.5 to 1 µg/kg intravenously as needed to maintain hemodynamic stability. Abdelhamid et al. [8] quantified total fentanyl consumption using incremental 50 µg doses, while Mostafa et al. [15] and Ashoor et al. [7] used weight-based boluses triggered by sympathetic stimulation.

For remifentanil-based protocols [9, 12–14], continuous infusion was employed and titrated intraoperatively. Doses ranged from 0.02 to 1.5 µg/kg/min, with adjustments based on real-time hemodynamic or anesthetic monitoring. Zengin et al. [13], for instance, implemented total intravenous anesthesia (TIVA) guided by bispectral index (BIS) and analgesia nociception index (ANI), ensuring precise control over anesthetic depth and analgesia. Similarly, Wang et al. [14] incorporated remifentanil into a balanced anesthesia regimen alongside sevoflurane and propofol, titrating infusions based on lean body weight.

Despite variations in opioid choice and monitoring tools, a common principle across all studies was the individualized titration of intraoperative analgesia to maintain physiologic parameters within 20% of baseline values.

**Fig. 4:**
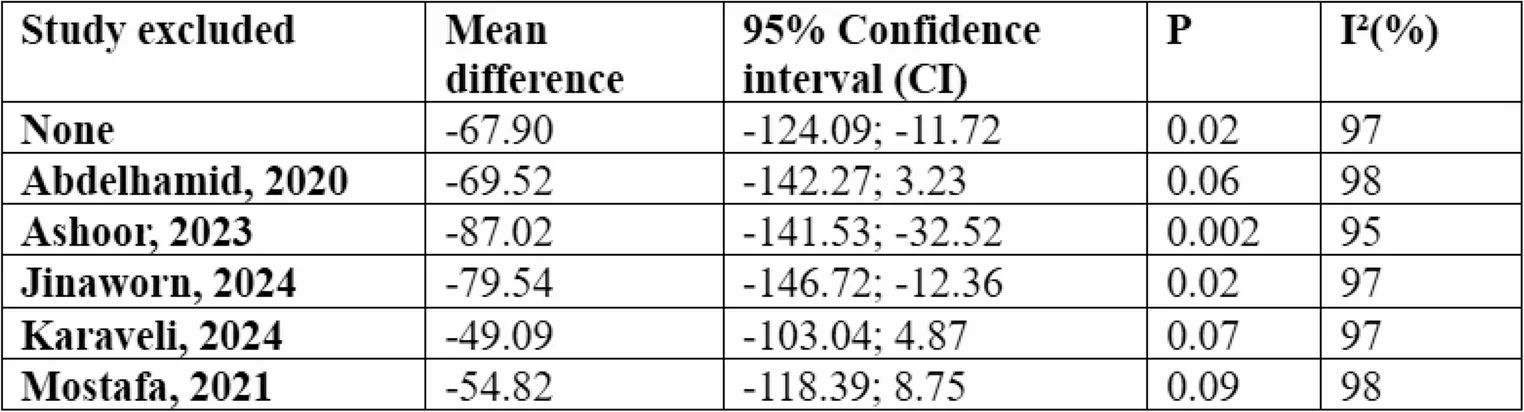
Leave-one-out - Intraoperative fentanyl consumption

To assess the robustness of the pooled result for intraoperative fentanyl consumption, a leave-one-out sensitivity analysis was conducted (Fig. 4). The individual exclusion of certain studies impacted the statistical significance of the finding specifically upon removing the studies by Abdelhamid 2020; Karaveli (2025) or Mostafa 2021 [8, 10, 15] the pooled mean difference lost its statistical significance, with the respective confidence intervals crossing the null value (MD −69.52, 95% CI −142.27 to 3.23, *P* = 0.06; MD −49.09, 95% CI −103.04 to 4.87, *P* = 0.07; and MD −54.82, 95% CI −118.39 to 8.75, *P*= 0.09). The exclusion of the remaining studies [7, 11] maintained a statistically significant result. Heterogeneity remained extremely high (≥ 95%) across all iterations of the analysis.

### NRS Score

**Fig. 5:**
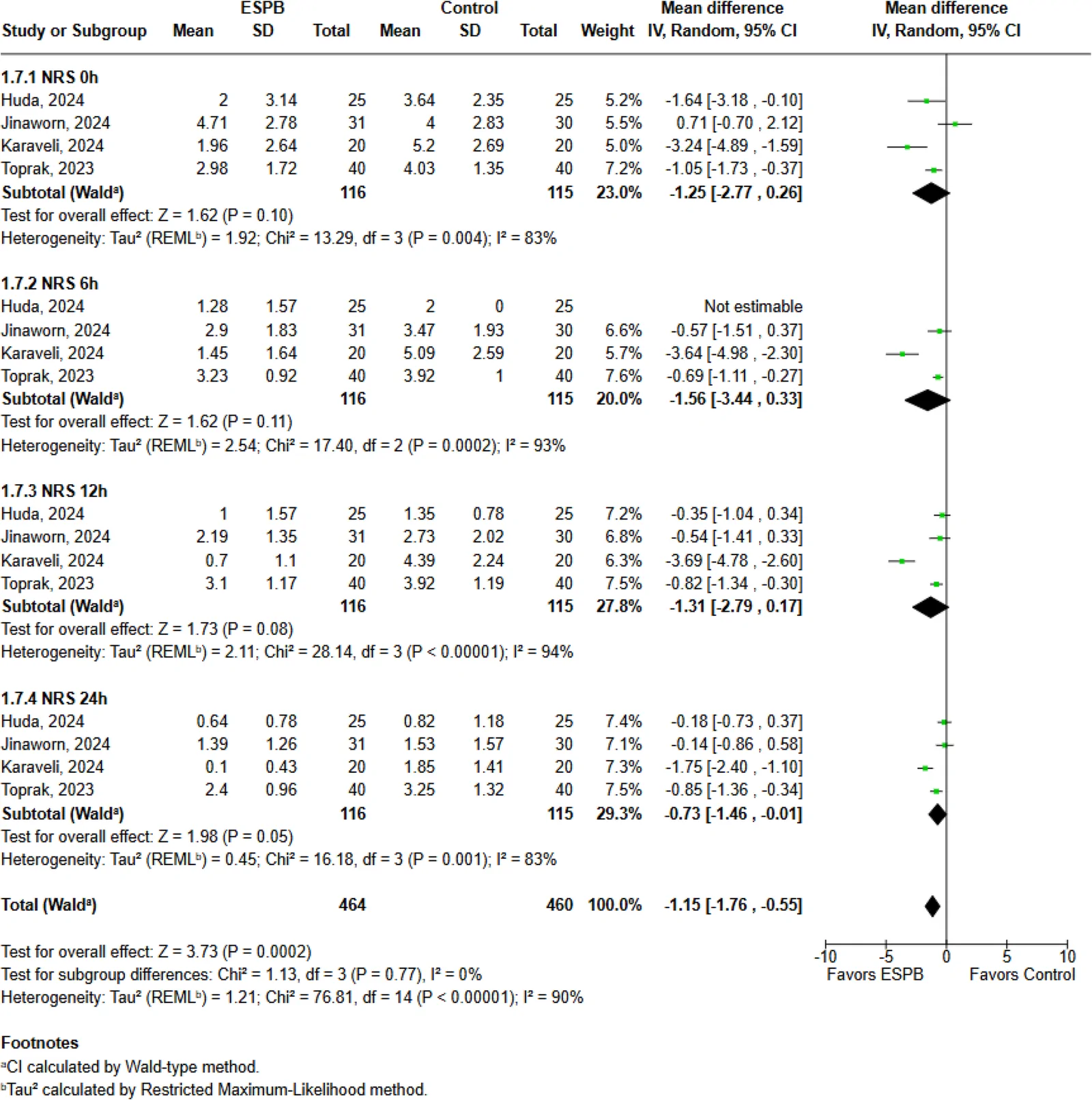
NRS score after surgery

We pooled the outcome of NRS (Numeric Rating Scale) for the postoperative pain scale of four studies. As shown in Fig. 5, we found that ESPB significantly reduced pain at rest up to 24 h after surgery (MD −1.15; 95% CI −1.76 to −0.55; *p* = 0.0002). Significant heterogeneity was initially observed in the pooled analysis (I² = 90%, *p* < 0.0001), prompting further exploration through subgroup analysis. Pain scores were stratified by postoperative time points at 0, 6, 12, and 24 h. No significant differences were found between subgroups (I² = 0%, *p* = 0.77), and each time point demonstrated either a favorable trend or statistically significant reduction in pain scores in the ESPB group compared to controls (Fig. 5).

A leave-one-out analysis for the Numeric Rating Scale (NRS) observed that the exclusion of the Karaveli (2025) study resulted in statistically significant mean differences at the 6 h (MD −0.67, *P* = 0.0006) and 12 h (MD −0.63, *P* = 0.0009) time points, while simultaneously reducing heterogeneity to 0% at both time points. (Table 2).

**Table 2:** Leave-one-out analysis for VAS score. VAS 0.5 h

NRS 0h				
Study excluded	**Mean ** **difference **	**95% ** **Confidence interval (CI) **	***P ***	**I²(%) **
None	−1.25	−2.77; 0.26	0.10	83
Huda, 2024 [13]	−1.15	−3.28; 0.98	0.29	89
Jinaworn, 2024 [11]	−1.81	−3.06; −0.56	0.005	66
Karaveli, 2025 [10]	−0.69	−1.94; 0.57	0.28	70
Toprak, 2023 [12]	−1.36	−3.61; 0.90	0.24	85
NRS 6h				
Study excluded	**Mean ** **difference **	**95% ** **Confidence interval (CI) **	***P ***	**I²(%) **
None	−1.56	−3.44; 0.33	0.11	93
Huda, 2024 [13]	−1.56	−3.44; 0.33	0.11	93
Jinaworn, 2024 [11]	−2.09	−4.98; 0.79	0.16	94
Karaveli, 2025 [10]	−0.67	−1.05; −0.29	0.0006	0
Toprak, 2023 [12]	−2.07	−5.07; 0.94	0.18	93
NRS 12h				
Study excluded	**Mean ** **difference **	**95% ** **Confidence interval (CI) **	***P ***	**I²(%) **
None	−1.31	−2.79; 0.17	0.08	94
Huda, 2024 [13]	−1.64	−3.57; 0.28	0.09	94
Jinaworn, 2024 [11]	−1.57	−3.57; 0.42	0.12	96
Karaveli, 2025 [10]	−0.63	−1.00; −0.26	0.0009	0
Toprak, 2023 [12]	−1.49	−3.59; 0.60	0.16	94
NRS 24h				
Study excluded	**Mean ** **difference **	**95% ** **Confidence interval (CI) **	***P ***	**I²(%) **
None	−0.73	−1.46; −0.01	0.05	83
Huda, 2024 [13]	−0.92	−1.81; −0.03	0.04	84
Jinaworn, 2024 [11]	−0.91	−1.79; −0.04	0.04	86
Karaveli, 2025 [10]	−0.42	−0.90; 0.05	0.08	50
Toprak, 2023 [12]	−0.69	−1.73; 0.35	0.19	87

*CI* confidence interval, *I²* Higgins' inconsistency statistic, *NRS* Numeric Rating Scale

###  VAS Score

**Fig. 6:**
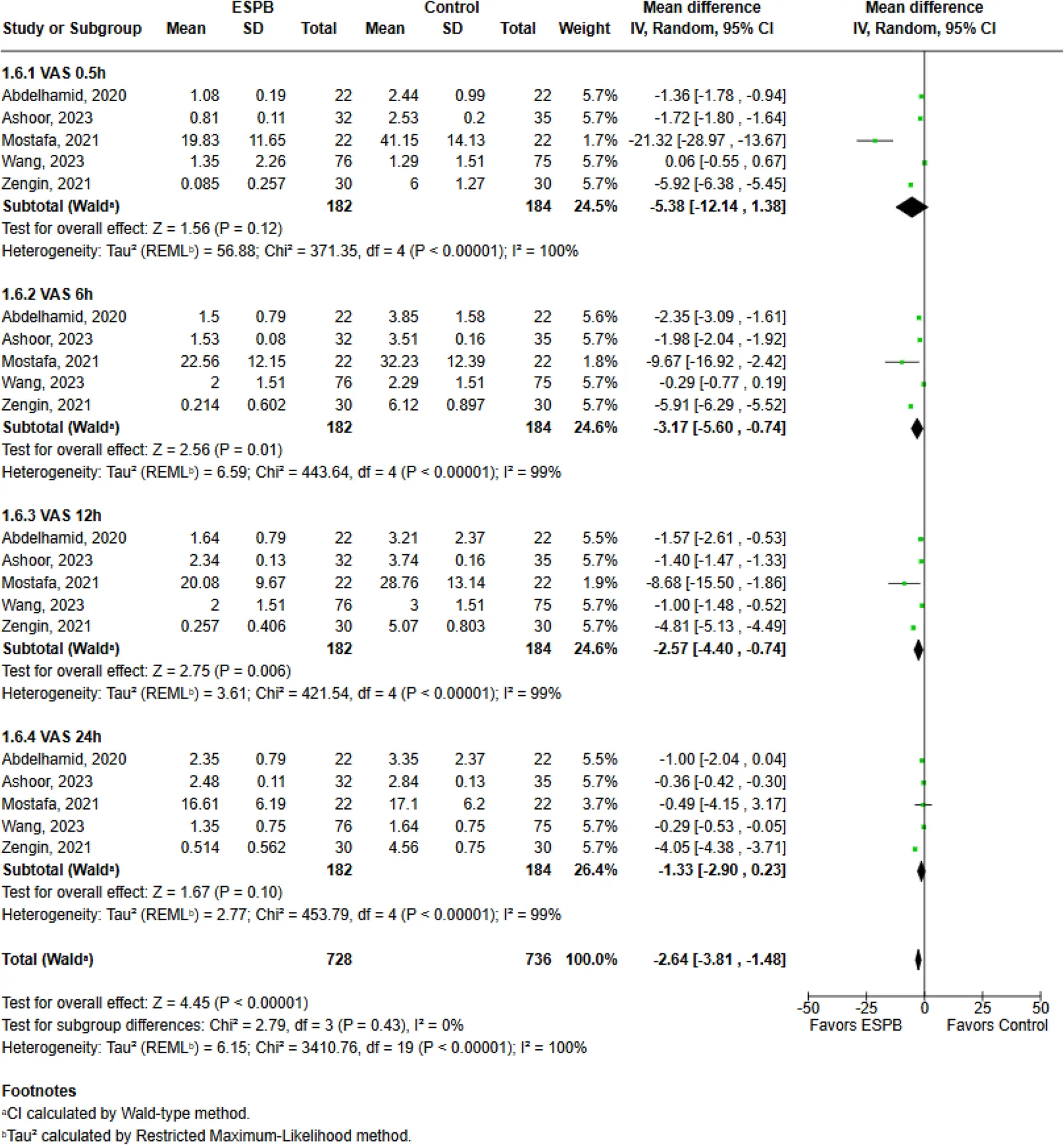
VAS score after surgery

VAS (Visual Analog Scale) score was evaluated in five studies to assess postoperative pain. We pooled data demonstrating ESPB reduced postoperative VAS score (MD −2.64; 95% CI −3.81 to −1.48; *p* < 0.00001). However, as shown in Fig. 6, this analysis exhibited high heterogeneity (I² = 100%; *p* < 0.0001), which was addressed using subgroup analysis. Pain scores were stratified by postoperative time points at 0.5, 6, 12, and 24 h. No significant differences were found between subgroups (I² = 0%, *p*= 0.43) and each time point demonstrated either a favorable trend or statistically significant reduction in pain scores in the ESPB group compared to controls.Zengin et al. was the outlier study in VAS 12 h and VAS 24 h.

For the Visual Analog Scale (VAS), the leave-one-out analysis (Table 3) also demonstrated that the exclusion of the Zengin 9 study caused a significant change in the result: at the 12 h and 24 h time points, the mean difference became highly significant (*P* < 0.00001) and heterogeneity decreased drastically (to 24% at 12 h and 0% at 24 h), indicating that this specific study was a potential source of variability.

**Table 3:** Leave-one-out analysis for NRS score. NRS 0 h

VAS 0.5h				
Study excluded	**Mean** **difference**	**95%** **Confidence interval (CI)**	***P***	**I²(%)**
None	−5.38	−12.14; 1.38	0.12	100
Abdelhamid, 2020 [8]	−6.61	−15.37; 2.14	0.14	100
Ashoor, 2023 [7]	−6.53	−15.36; 2.30	0.15	100
Mostafa, 2021 [15]	−2.24	−4.75; 0.28	0.08	100
Wang, 2023 [14]	−6.93	−15.29; 1.42	0.10	100
Zengin, 2021 [9]	−5.48	−14.63; 3.67	0.24	100
VAS 6 h				
Study excluded	**Mean** **difference**	**95%** **Confidence interval (CI)**	***P***	**I²(%)**
None	−3.17	−5.60; −0.74	0.01	99
Abdelhamid, 2020 [8]	−3.59	−6.98; −0.21	0.04	100
Ashoor, 2023 [7]	−3.69	−7.03; −0.36	0.03	99
Mostafa, 2021 [15]	−2.63	−4.95, - 0.32	0.03	99
Wang, 2023 [14]	−3.97	−6.52; −1.42	0.002	99
Zengin, 2021 [9]	−1.79	−3.16; −0.43	0.01	96
VAS 12h				
Study excluded	**Mean ** **difference **	**95% ** **Confidence interval (CI) **	***P ***	**I²(%) **
None	−2.57	−4.40; −0.74	0.006	99
Abdelhamid, 2020 [8]	−2.99	−5.51; −0.48	0.02	99
Ashoor, 2023 [7]	−3.05	−5.54; −0.57	0.02	98
Mostafa, 2021 [15]	−2.21	−3.96; −0.46	0.01	99
Wang, 2023 [14]	−3.12	−5.43; −0.81	0.008	99
Zengin, 2021 [9]	−1.33	−1.60; −1.07	<0.00001	24
VAS 24h				
Study excluded	**Mean ** **difference **	**95% ** **Confidence interval (CI) **	***P ***	**I²(%) **
None	−2.57	−4.40; −0.74	0.006	99
Abdelhamid, 2020 [8]	−2.99	−5.51; −0.48	0.02	99
Ashoor, 2023 [7]	−3.05	−5.54; −0.57	0.02	98
Mostafa, 2021 [15]	−2.21	−3.96; −0.46	0.01	99
Wang, 2023 [14]	−3.12	−5.43; −0.81	0.008	99
Zengin, 2021 [9]	−1.33	−1.60; −1.07	<0.00001	24

*CI* confidence interval, *I²* Higgins' inconsistency statistic, *VAS* Visual Analog Scale

### Total Opioid Consumption

Due to the heterogeneity of opioid types (morphine, tramadol, oxycodone, pethidine, nalbuphine), timing of administration, and PCA parameters, initially, a direct quantitative synthesis using mean difference was not feasible. However, qualitative synthesis supports the fact that ESPB is associated with reduced opioid requirements in morphine-based protocols.

**Fig. 7:**
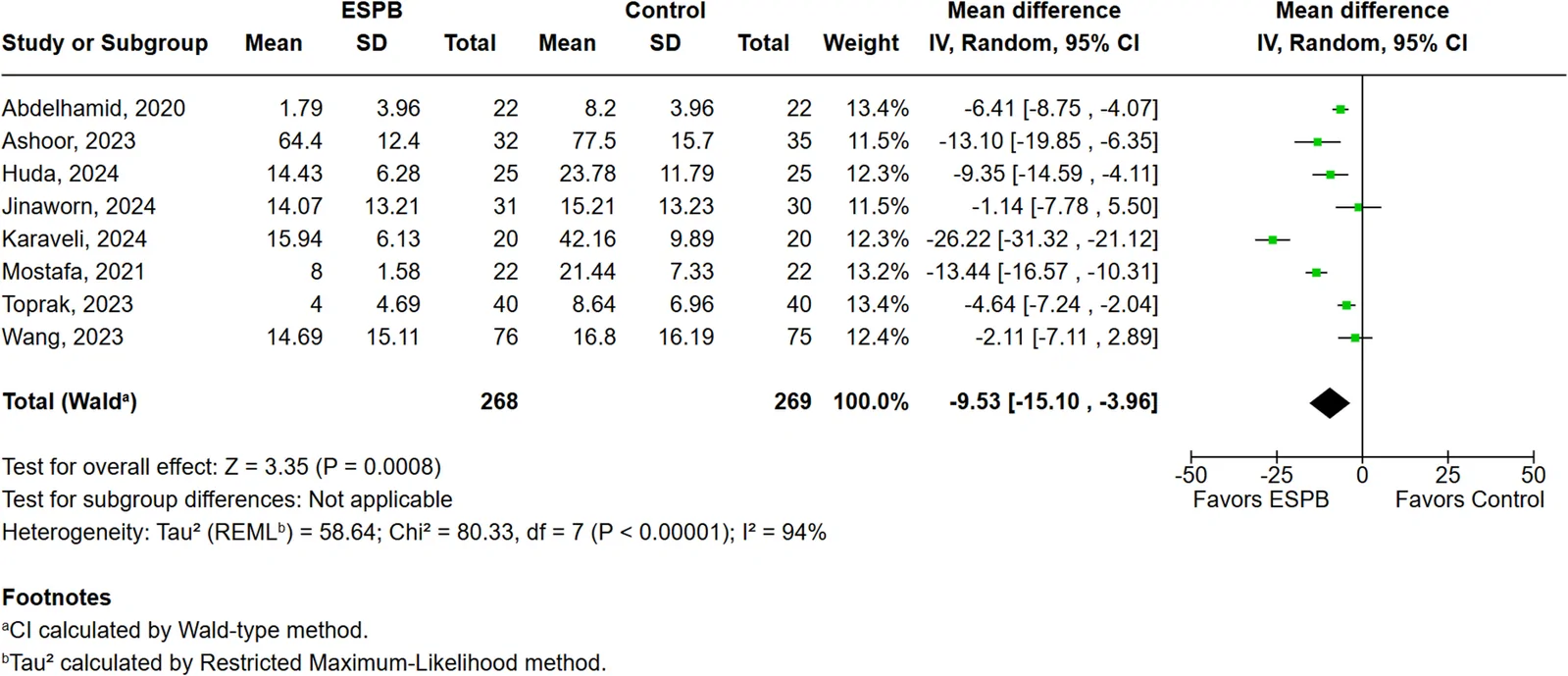
Total opioid consumption

For the purposes of meta-analysis, all opioid doses were standardized and converted to morphine equivalents to allow consistent comparison across studies. We pooled data on total opioid consumption from eight studies and found that ESPB significantly reduced total opioid consumption (MD −9.53, 95% CI −15.10 to −3.96, *p* = 0.0008) (Fig. 7). A high heterogeneity (I²=94%, *p* < 0.00001) was observed, attributed to differences in analgesic protocols and opioid types used across the trials, and no single study was responsible for the heterogeneity.

Across the included studies, most authors employed a multimodal analgesia strategy, with IV paracetamol (1 g) consistently administered either before extubating or at the end of surgery in nearly all protocols. Several studies (Ashoor, Karaveli, Toprak, Zengin) incorporated tramadol as part of baseline or rescue analgesia, while others (Jinawork, Huda, Mostafa, Toprak) utilized nonsteroidal anti-inflammatory drugs (NSAIDs) such as parecoxib, lornoxicam, tenoxicam, or flurbiprofen to enhance pain control.

Patient-controlled analgesia (PCA) systems were employed in multiple studies, with agents including morphine (Huda, Jinawork, Mostafa) and nalbuphine (Ashoor), typically combined with bolus-only regimens and individualized lockout intervals. Wang et al. uniquely administered oxycodone and flurbiprofen ester via PCA. In several protocols, additional agents such as dexamethasone and ondansetron were administered perioperatively for anti-inflammatory and antiemetic purposes.

Rescue analgesia thresholds were relatively consistent, with additional opioids (e.g., morphine, tramadol, pethidine) provided when pain scores exceeded predefined cutoffs, typically NRS ≥ 4 or VAS ≥ 3–5.

Regarding the leave-one-out analysis for total opioid consumption the effect remained significant for total opioid consumption even after the individual exclusion of each of the listed studies (including Abdelhamid, 2020; Ashoor, 2023; Huda 2024; Jinaworn, 2024; Karaveli, 2025; Mostafa, 2021; Toprak, 2023 and Wang, 2023, [7, 8, 10–15] confirming the robustness of this conclusion (Table 4).

**Table 4:** Leave-one-out analysis for total opioid consumption

Study excluded	Mean difference	95% Confidence interval (CI)	*P*	I²(%)
None	−9.53	−15.10; −3.96	0.0008	94
Abdelhamid, 2020 [8]	−10.02	−16.43; −3.60	0.002	93
Ashoor, 2023 [7]	−9.07	−15.36; −2.78	0.005	95
Huda, 2024 [13]	−9.56	−16, −3.13	0.004	95
Jinaworn, 2024 [11]	−10.62	−16.50; −4.75	0.0004	94
Karaveli, 2025 [10]	−7.21	−10.70; −3.72	< 0.0001	82
Mostafa, 2021 [15]	−8.95	−15.29; −2.60	0.006	94
Toprak, 2023 [12]	−10.29	−16.56; −4.02	0.001	93
Wang, 2023 [14]	−10.58	−16.56; −4.61	0.0005	94

*CI* confidence interval, *I²* Higgins’ inconsistency statistic

### Respiratory Complications

**Fig. 8:**
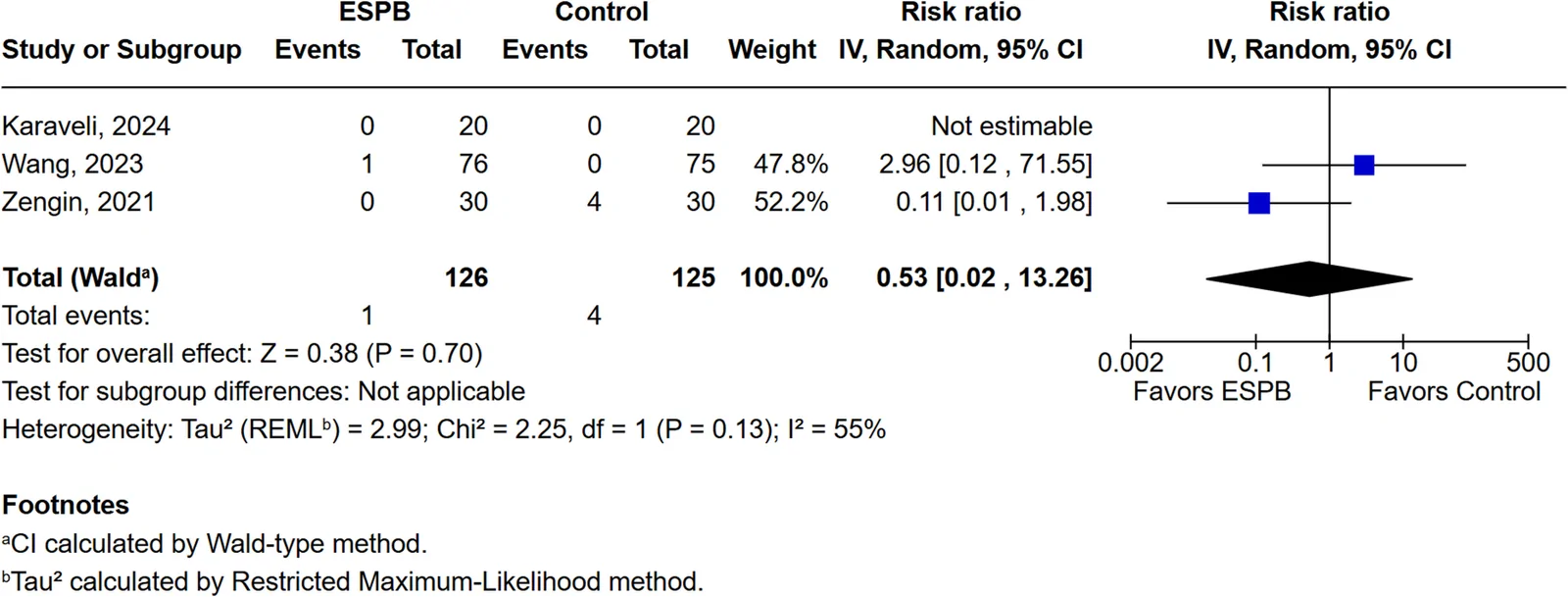
Respiratory complications

We pooled data from three studies and found no significant differences in respiratory complications between ESPB and the control group (RR 0.53, 95% CI 0.02 to 13.26, *p* = 0.70) (Fig. 8).

### PONV

**Fig. 9:**
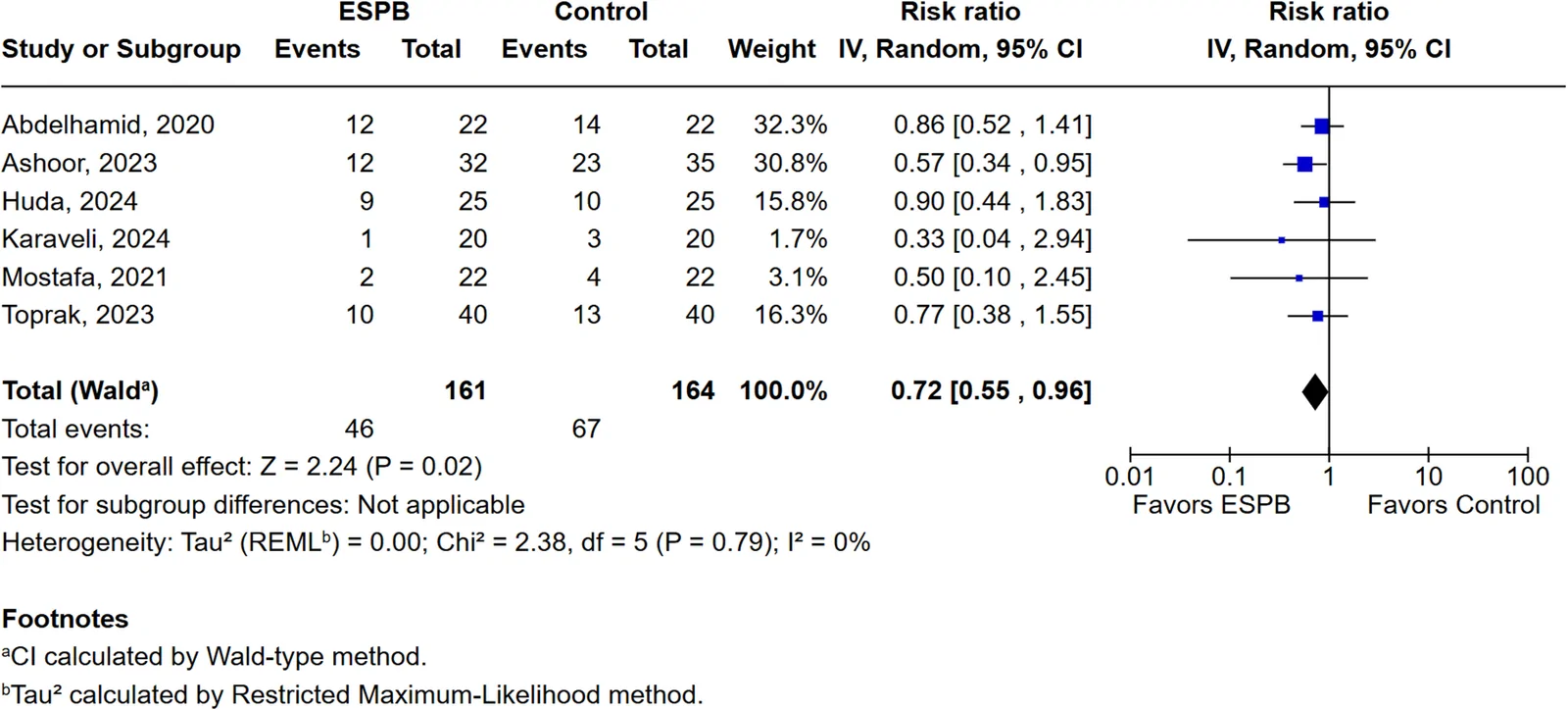
PONV after surgery

We pooled events of postoperative nausea and vomiting (PONV) in 6 studies. Figure 9 shows ESPB as significant to reduce the incidence of PONV compared to the control group (RR = 0.72; 95% CI 0.55 to 0.96; *p* = 0.02). Heterogeneity was low (I²= 0.0%; *p* = 0.79) (Fig. 9).

### Risk of Bias

In the risk of bias analysis regarding the randomization process (D1), the study by Huda et al. used sealed opaque envelopes for participant allocation, which is considered acceptable if there is a clear and robust description of the method. However, the study does not specify whether the envelopes were numbered sequentially, whether the process was supervised, or if the person opening the envelopes could anticipate the allocation, which raises uncertainties about the concealment of randomization. The study by Toprak et al. claims randomization occurred, but it does not describe the method used to generate the random sequence or the allocation concealment mechanism, which prevents verification of the process’s integrity. Similarly, Jinaworn et al. does not provide details on the sequence generation and, although it mentions the use of allocated envelopes, there is insufficient information to guarantee that the allocation was adequately concealed. Moreover, the envelope was opened by the anesthesiologist responsible for the intervention, which increases the potential risk of bias.

Regarding deviations from the intended intervention (D2), the studies by Toprak, Huda, and Karaveli did not include a placebo group, and there was no blinding of the anesthesiologists, which presents a potential performance bias risk. The study by Jinaworn also had incomplete blinding, as the anesthesia team was aware of the allocation. Similarly, the study by Zengin did not use a placebo group and allowed the possibility that the attending team knew which intervention was being applied. Concerning bias due to missing outcome data (D3), none of the studies showed significant data losses or relevant omissions that could compromise the validity of the outcomes assessed.

**Fig. 10:**
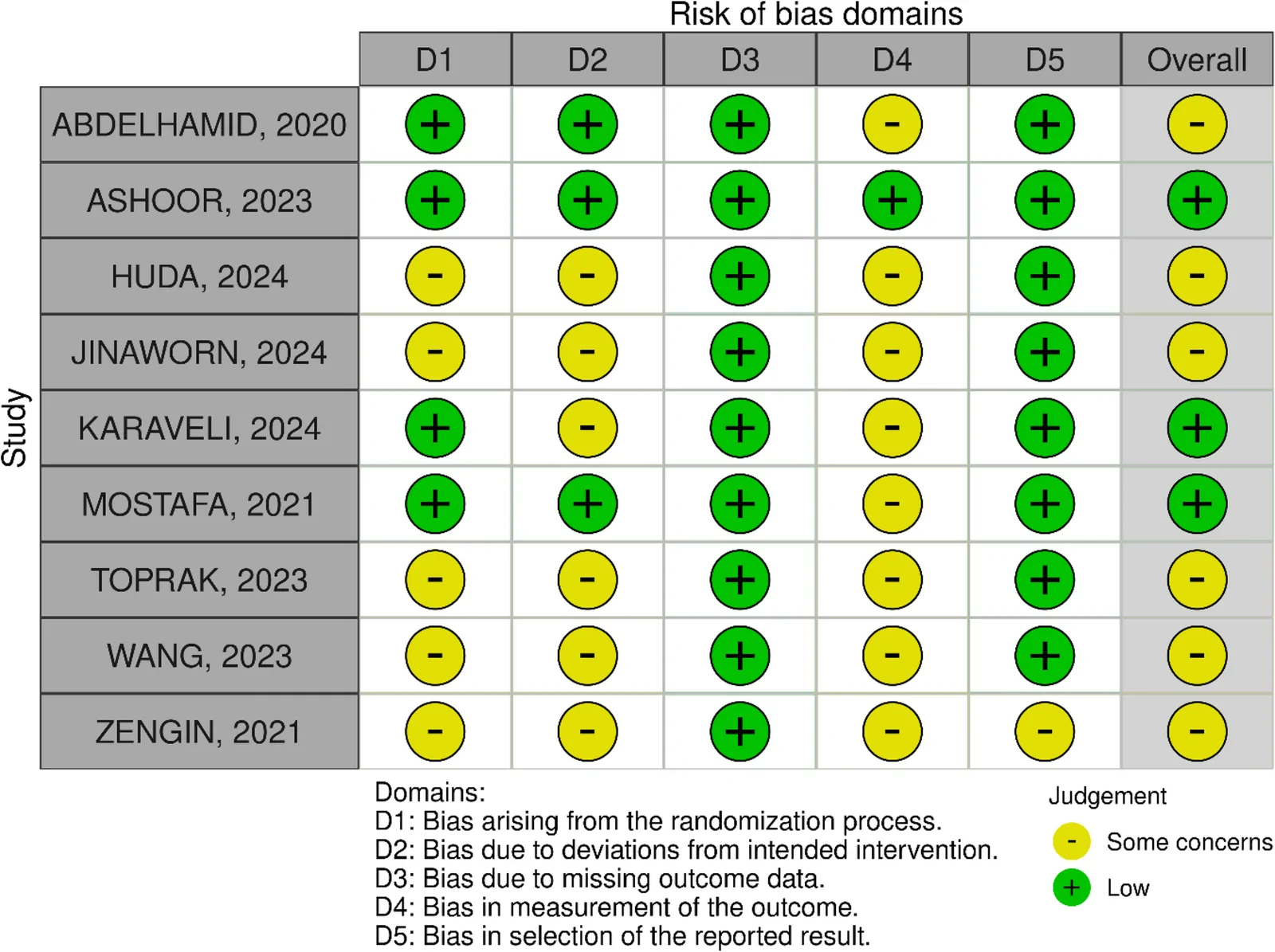
Critical appraisal of individual studies according to the Cochrane Collaboration’s tool for assessing risk of bias in randomized trials

In the assessment of bias in outcome measurement (D4), it was noted that the studies used subjective pain scales such as the VAS (Visual Analog Scale) and NRS (Numeric Rating Scale), which can introduce measurement bias, especially when assessors are not blinded. However, the study by Ashoor et al. employed adequate measures to mitigate this risk by using blinded assessors to apply the VAS scale. Finally, regarding bias in the selection of the reported outcome (D5), only the study by Zengin et al. raised concerns about selective reporting. Although the study focused on opioid consumption and pain outcomes, it did not mention any adverse events related to the anesthetic technique used, which may indicate the omission of unfavorable results and, therefore, a risk of selective reporting bias. Figure 10 summarized the risk of bias analysis.

## Discussion

This systematic review and meta-analysis included nine randomized controlled trials comprising a total of 553 patients, including 459 who underwent sleeve gastrectomy and 94 who underwent bypass surgery. Our analysis showed a significant reduction in postoperative pain across two distinct assessment scales, along with favorable intraoperative consumption of both fentanyl and remifentanil. Moreover, postoperative opioid requirements and nausea were significantly reduced in the ESPB group, without any differences in respiratory complications.

The findings of this meta-analysis have significant clinical implications, particularly for integration into Enhanced Recovery After Surgery (ERAS) protocols for bariatric patients. A central principle of ERAS is the implementation of multimodal, opioid-sparing analgesia to mitigate the adverse effects that delay recovery [16–21]. Our results demonstrate that ESPB is an effective component of such a strategy, evidenced by a significant reduction in both intraoperative fentanyl and total postoperative opioid consumption. This opioid-sparing effect is critical, as it translates directly to a decreased incidence of opioid-related adverse effects. Crucially, this review found that ESPB significantly reduces postoperative nausea and vomiting (PONV), a primary factor impeding early oral intake and mobilization. By attenuating PONV and improving pain control, the ESPB can facilitate adherence to key ERAS milestones, thereby promoting faster recovery and potentially reducing hospital length of stay.

With advancements in anesthetic blocks, a comprehensive study was necessary to emphasize the importance of effective pain control, opioid reduction, and the prevention of complications such as nausea, vomiting, and respiratory issues. A previous meta-analysis was published, but it had significant limitations that compromised its conclusions[22]. The previous meta-analysis (1) was not registered in the PROSPERO database; (2) included non-bariatric studies such as Tang et al., which examined cognitive outcomes in hip surgery patients and is only indexed on Google Scholar; (3) the review incorrectly grouped comparisons of ESPB versus “other blocks,” while in fact, Abdelhamid et al. compared it with subcostal TAP, Ashoor et al. with QLB, Elshazly et al. with TAP, and Wang et al. with paravertebral block. (4) the authors rated the risk of bias as “low” across nearly all domains; (5) they aggregated different pain scales (NRS and VAS), which are not interchangeable and should have been standardized using the standard mean difference (SMD) to allow meaningful comparison; (6) crucially, no sensitivity analyses were performed in the previous meta-analysis to account for heterogeneity. The present review seeks to address these gaps and provide a more precise understanding of how enhanced pain management techniques can improve patient recovery.

Several studies have demonstrated the potential benefits of preoperative anesthetic blocks in reducing postoperative pain and complications. Two recent meta-analyses further support this evidence in different surgical contexts and age groups [23, 24]. One systematic review and meta-analysis assessed the efficacy of ESPB in adult patients undergoing unilateral inguinal hernia repair [23]. Across four randomized controlled trials with 181 patients, the ESP block significantly reduced pain scores at 6 and 12 h postoperatively and was associated with a lower incidence of nausea and vomiting. Another meta-analysis evaluated the role of ESPB in pediatric patients undergoing cardiac surgery via sternotomy [24]. The review included five studies (three RCTs and two cohorts) with 328 children; the ESPB significantly reduced cumulative opioid consumption up to 48 h postoperatively. These findings reinforce the growing evidence for the use of ESPB across age groups and surgical settings, particularly in minimizing opioid use and enhancing postoperative recovery.

Erector spinae plane (ESP) blocks can be challenging in obese patients due to increased adipose tissue affecting ultrasound visualization and the depth of the block. However, studies suggest that ESPB is still feasible and effective in this population for pain management, particularly in bariatric surgery [25].

Persistent opioid use following surgery is not only clinically harmful but also economically burdensome. A large-scale study involving over 3 million patients across commercial, Medicare, and Medicaid cohorts found that new persistent opioid use occurred in up to 7.1% of inpatients and 6.4% of outpatients, even among previously opioid-naive individuals. These patients consistently presented with higher comorbidity burdens and required more frequent use of health services, including hospital readmissions, emergency care, and paramedic services, within one year postoperatively. More critically, healthcare costs were significantly higher for those who developed persistent opioid use. After adjusting for baseline factors, total one-year healthcare costs were 2 to 3 times greater in these patients across all payer groups. For example, commercial inpatients with persistent use incurred $29,499 vs. $11,798, and Medicaid outpatients $12,381 vs. $6,784 (*P*< 0.001 for all comparisons) [19]. These findings underscore the need to minimize opioid exposure whenever possible—not only to reduce adverse effects like nausea, sedation, and respiratory depression, but also to contain long-term healthcare costs and improve postoperative outcomes [26]. Regional techniques and opioid-sparing strategies such as ESPB should be prioritized in perioperative care.

Our study presents strong findings in the field of pain control and opioid use, highlighting important advances in postoperative care for bariatric patients. In our study, subgroup analyses and leave-one-out analysis were conducted to validate the data. One factor that undermines the previous meta-analysis is that opioid consumption was assessed with different anesthetic drugs, which have varying bioequivalence. In our meta-analysis, we standardized the intraoperative use of remifentanil, a continuous infusion, compared to fentanyl, which is administered periodically. We also standardized the postoperative opioid doses to ensure equivalence with morphine.

Despite these findings, the conclusions of this meta-analysis must be interpreted in the context of several limitations. First, significant methodological heterogeneity was observed across primary outcomes, including total opioid consumption (I²=94%), NRS scores (I²=90%), and VAS scores (I²=100%). This variability is likely attributable to the diverse analgesic protocols, types of opioids utilized, differing PCA regimens, variations in local anesthetic dose and volume, differences in the timing of block administration (before versus after surgical incision), and operator experience. Because only nine studies were available, formal meta-regression to explore these potential sources of heterogeneity was not feasible. Second, the risk of bias analysis identified methodological concerns; a notable issue is the lack of participant and personnel blinding in several studies, which introduces a significant risk of bias performance, particularly for subjective outcomes like pain scores. Specifically, concern arises with the outcome measurement, particularly the visual pain scale (D4), which is entirely subjective and may be prone to bias. It could have been measured after the administration of medications, at a time when the patient was more relaxed, for example, which could influence the results. These factors should be interpreted with caution. Other limitations are worth mentioning, particularly in relation to the randomization process (D1). The studies by Toprak et al., Jinaworn et al., Huda et al., and Zengin et al. did not fully describe the randomization method, which raises some concerns. Regarding deviations from the intended intervention (D2), the studies by Toprak et al., Huda et al., and Karaveli et al. showed an absence of a placebo group. There was no blinding of the anesthesiology team; Jinaworn et al. also presented incomplete blinding - the anesthesiology team was not blinded. At the same time, the study by Zengin et al. also did not present a placebo group, and the possibility of knowing the intervention. Finally, the generalizability of these findings may be limited, as the pooled patient population was predominantly composed of individuals undergoing laparoscopic sleeve gastrectomy (*n* = 459), with a much smaller subset undergoing gastric bypass (*n* = 94). This imbalance restricts the confident extrapolation of these results to the gastric bypass population, which may present distinct analgesic requirements.

Nevertheless, this represents the most statistically robust systematic review and meta-analysis to date on the topic, as it incorporates subgroup analyses and accounts for anesthetic protocol variability through dose standardization and clinical matching. This methodological rigor enhances the reliability of the pooled estimates and strengthens the overall validity of the findings.

## Conclusion

This meta-analysis suggests that ultrasound-guided erector spinae plane block is an effective adjunct for pain management in bariatric surgery, significantly reducing intraoperative and postoperative opioid consumption, PONV, and pain scores. While no significant effect was observed on respiratory complications, ESPB appears to enhance multimodal analgesia strategies.

## Supplementary Information

Below is the link to the electronic supplementary material.

Supplementary Material 1 (DOCX 15.3 KB)

## Data Availability

No datasets were generated or analysed during the current study.
