# Supplementary material for: Erector Spinae Block in Bariatric Surgery: a Systematic Review and Meta-Analysis of Randomized Controlled Trials
Source: Obes Surg. 2026 Sep 22;36(10):5528–42. doi: 10.1007/s11695-026-08935-4 (PMC13645966; doi:10.1007/s11695-026-08935-4)
Supplement: Supplementary file 1 [file 11695_2026_8935_MOESM1_ESM.docx]

**Supplementary material**

**Search strategy**

# PUBMED

("bariatric surgery" OR "Bariatric Surgery"[Mesh] OR "sleeve" OR "gastrectomy" OR

"Gastrectomy"[Mesh] OR "gastric bypass" OR "Gastric Bypass"[Mesh]) AND

("ultrasound-guided" OR "erector spinae plane block" OR "ESP block") AND (randomized controlled trial[pt] OR controlled clinical trial[pt] OR clinical trials as topic[mesh:noexp] OR trial[ti] OR random*[tiab] OR placebo*[tiab])

# EMBASE

('bariatric surgery'/exp OR 'bariatric surgery' OR 'sleeve' OR 'gastrectomy'/exp OR

'gastrectomy' OR 'gastric bypass'/exp OR 'gastric bypass') AND ('ultrasound-guided' OR

'erector spinae plane block'/exp OR 'erector spinae plane block' OR 'esp block') AND 'controlled clinical trial'/exp

# COCHRANE

("bariatric surgery" OR "sleeve" OR "gastrectomy" OR "gastric bypass") AND

("ultrasound-guided" OR "erector spinae plane block" OR "ESP block") AND (randomized controlled trial)
